# OTUD7B is a new deubiquitinase targeting p53

**DOI:** 10.7150/thno.103012

**Published:** 2025-01-13

**Authors:** Caoyuan Ding, Leixi Cao, Ruijie Wang, Qichen Wu, Mengfan Li, Jinjing Zhang, Rick F. Thorne, Jinming Li, Jianli Ma, Mian Wu, Shundong Cang

**Affiliations:** 1 Translational Research Institute, People's Hospital of Zhengzhou University, 450003 Zhengzhou, Henan, China.; 2 Department of Pathophysiology, School of Basic Medical Sciences, Zhengzhou University, 450001 Zhengzhou, Henan, China.; 3 Department of Radiation Oncology, Harbin Medical University Cancer Hospital, 150081 Harbin, Heilongjiang, China.

**Keywords:** OTUD7B, Deubiquitination, p53, HCC, Apoptosis.

## Abstract

**Rationale:** The tumor suppressor p53 safeguards against cellular transformation, with its expression regulated by diverse post-translational modifications (PTMs). While polyubiquitination by Mdm2 principally drives its proteasomal degradation, the identity of p53 deubiquitinases (DUBs) remains less well defined. This study investigates the role of the deubiquitinase enzyme OTUD7B in hepatocellular carcinoma (HCC), where it is notably downregulated and proposed to function as a tumor suppressor.

**Methods:** Mass spectrometry screening of immunoprecipitates from HCC cells was used to identify OTUD7B-binding proteins. Co-immunoprecipitation assays with endogenous, ectopic, and mutant forms of OTUD7B and p53 assessed binding interactions and p53 polyubiquitination levels, respectively. Regulatory mechanisms were explored via luciferase reporter and chromatin immunoprecipitation (ChIP) assays. OTUD7B function was evaluated in vitro and in xenograft models using shRNA knockdown, overexpression, and CRISPR-Cas9 knockout. OTUD7B expression in normal and HCC tissues was analyzed by immunohistochemistry and immunoblotting.

**Results:** We identified p53 as a binding partner of OTUD7B, confirming interactions with both wild-type and mutant p53 in HCC cells. OTUD7B was shown to remove lysine-linked polyubiquitin chains in p53, including those mediated by Mdm2, thereby stabilizing p53 by inhibiting its proteasomal degradation. Overexpression of OTUD7B suppressed growth in HCC cultures and xenografts through p53-dependent mitochondrial apoptosis, marked by PUMA and BAX induction. Conversely, OTUD7B knockdown promoted tumor growth. These effects were absent in p53-null or CRISPR-knockout cells, underscoring p53 as a key OTUD7B substrate. Additionally, OTUD7B expression was found to be transcriptionally repressed via p53-dependent mechanisms. Bioinformatics and *ex vivo* analysis revealed a positive correlation between OTUD7B and p53 protein levels in HCC tissues.

**Conclusion:** OTUD7B plays a critical role in stabilizing both wild-type and mutant p53 in HCC cells, with its expression regulated through a mutual feedback loop involving p53. By inhibiting cell growth, OTUD7B exhibits tumor-suppressive properties, underscored by its atypical downregulation in patient tissues and its positive correlation with p53 expression. These findings highlight the clinical significance of OTUD7B and position it as a promising therapeutic target for modulating the p53 pathway in HCC.

## Introduction

Protein ubiquitination is one of the most extensively studied PTMs, so named because of its universal presence in all organisms where it plays a crucial regulatory role in numerous signaling pathways 1-3. Beyond the enzyme systems responsible for the attachment of ubiquitin, those which remove or cleave ubiquitin molecules attached to proteins remain equally critical components of the ubiquitin-proteasome system (UPS). Importantly, perturbations in the expression of such de-ubiquitination enzymes are associated with every recognized hallmark and enabling characteristic of cancer 4. Among these, OTUD7B, also known as Cezanne, is a member of the ovarian tumor protease superfamily (OTU) of DUBs. OTU DUB functions have been linked with various important signaling cascades associated with the development and progression of tumors. For example, OTUB1 inhibits RNF168-mediated polyubiquitination, thereby enhancing DNA damage responses 5; OTUD3 deubiquitinates and stabilizes PTEN to inhibit the activation of Akt signaling 6, and directly deubiquitinates and stabilizes p53 7; OTULIN inhibits cell death and inflammation by deubiquitinating LUBAC 8, also mediating Met1 deubiquitination, thus negatively regulating downstream NF-κB signaling 9; while A20/TNFaIP3 negatively regulates the NF-κB signaling pathway 10.

Like other OTU members including A20, OTUD7B is a multifunctional enzyme that is structurally composed of a deubiquitinating activity domain (OTU), a ubiquitin-associated domain (UBA), and a zinc finger domain. Notably, OTUD7B has been shown to play roles in regulating the cell cycle, the differentiation of neural progenitor cells, inflammatory responses, mucosal immunity, tumors and other diseases associated with the non-classical NF-κB pathway, acting to catalyze the deubiquitination of different substrates including cyclin B 11, EGFR 12, GβL 13, Sox2 14, Zap70 15, and TRAF3 16. Previous studies indicate that OTUD7B is widely distributed among human cancers where it is often highly expressed in gastric cancers 17, pancreatic cancers 18, 19, breast cancers 13, 20, 21, and lung cancers 16, 22, 23, serving to exert cancer-promoting effects. For example, high OTUD7B expression in breast cancer acts to deubiquitinate LSD1, in turn activating histone deacetylase complexes to promote metastasis through epigenetic effects 19. However, other research has revealed a contrastingly lower differential expression of OTUD7B in HCC compared to its occurrence in other tumor types 24, 25. The latter immunohistochemical study by Wang and colleagues showed stage-dependent decreases in OTUD7B protein expression, moreover, reporting that its downregulation was associated with faster relapse and death 25. These studies propose that OTUD7B suppresses tumor progression in HCC although the underlying molecular mechanisms and cause of OTUD7B downregulation in HCC are largely unknown.

HCC is one of the most common malignant tumors, ranking sixth in incidence and second in mortality rate among cancers worldwide. Moreover, its incidence is on the rise each year, posing a significant global health issue 26. For patients with advanced liver cancer, targeted therapy is the treatment of choice, yet the rate of disease control remains low 27. Of further relevance to this report is the *TP53* gene encoding the p53 protein discovered by David Lane's team in 1979, arguably the most crucial tumor suppressor gene known 28, variously dubbed as 'the guardian of the genome' and 'the Death Star' 29, 30. One of the primary mechanisms by which p53 functions is as a transcription factor 31, where it can positively and negatively regulate the expression of hundreds of different response genes, thereby participating in the regulation of virtually all biological processes, including the cell cycle, cell migration, apoptosis, cell aging, differentiation, metabolism, and autophagy, among others 32. To maintain their growth and survival, tumors employ various strategies to disarm p53, such as mutations in *TP53*. Mutations in the *TP53* tumor suppressor gene represent some of the most common genetic alterations in cancer, occurring in about half of all cancer cases including HCC 33. Indeed, in HCC, various viruses and chemicals induce *TP53* mutations in the liver 34, for example, Aflatoxin B1 can induce *TP53* mutations 35, promoting tumor growth and inhibiting apoptosis mediated by wildtype p53 to increase cancer cell survival 36, 37. Thus, the regulation of p53 expression and function plays an important role in HCC, with knowledge of the cell factors involved in this process providing valuable clues for new therapeutic approaches. In this regard, the deubiquitinating enzymes regulating p53 protein stability in HCC are still poorly understood.

In this report, we identified OTUD7B as a novel p53 deubiquitinating enzyme which functions in HCC and likely in other cancer types. Restoring the diminished levels of OTUD7B serves to inhibit HCC cell growth both *in vitro* and* in vivo*. The latter results from stabilizing p53 expression, which also impacts the expression and function of Mdm2 in a p53-dependent manner, disrupting the ability of Mdm2 to regulate p53 levels. Moreover, these findings reflect more widespread effects on both the transcriptional and non-transcriptional activities of p53, with the growth inhibitory effects of OTUD7B primarily associated with the promotion of apoptosis but not other forms of cell death. Interestingly, OTUD7B can recognize mutant forms of p53 although its actions are lost in HCC cells lacking p53 expression. Together these findings propose OTUD7B as a potential therapeutic target for liver cancer.

## Results

### Identification of p53 as an OTUD7B-interacting protein in HCC

**Figure 1:**
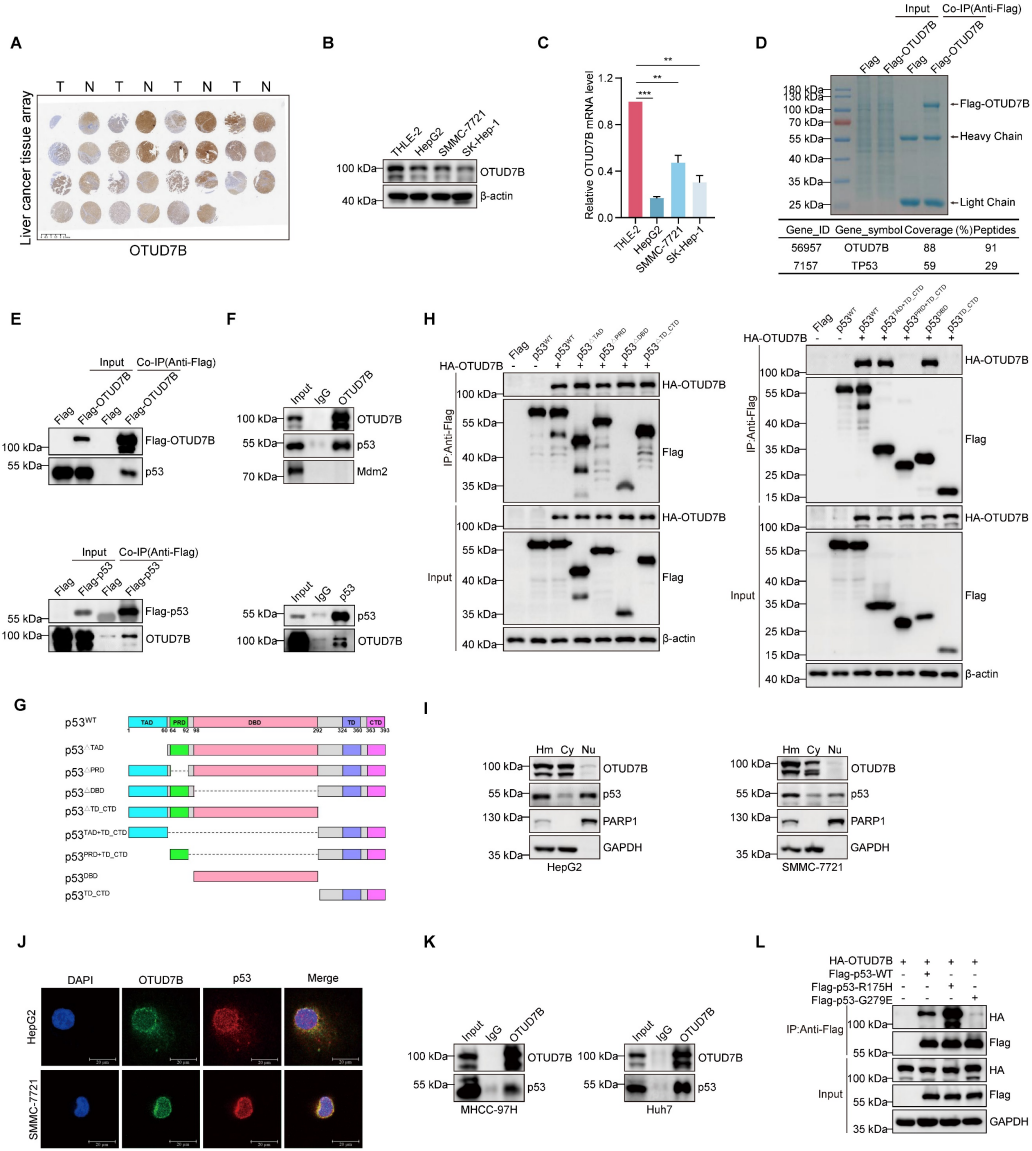
** Identification of OTUD7B as a p53 interacting protein. A.** IHC staining against OTUD7B in a tissue microarray consisting of 15 matched (left-right) pairs of hepatocellular carcinoma and normal liver tissues. **B-C.** Comparative OTUD7B protein (A) and mRNA (B) expression levels among different hepatic carcinoma cell lines analyzed by Western blotting and qPCR, respectively. **D.** Coomassie Brilliant Blue staining of proteins pulled down in association with OTUD7B (upper). HEK293T cells were transfected with either Flag vector alone or Flag-tagged OTUD7B and total protein extracts subjected to anti-Flag antibody IP before characterization of specific bands by mass spectrometry. OTUD7B along with p53 was recovered with high peptide coverage (lower). **E.** HEK293T cells were transfected with Flag-OTUD7B (left) and Flag-p53 (right) constructs were subject to IP and immunoblotting with anti-Flag, p53 and OTUD7B antibodies as indicated. Endogenous p53 and OTUD7B were enriched in the Flag immunoprecipitates. **F.** The co-immunoprecipitation experiment in (E) was repeated to analyze interactions between endogenous OTUD7B and p53 in HepG2 cells. OTUD7B reciprocally co-precipitated with p53 but not Mdm2. **G-H.** Domain structure of p53 protein and deletion mutant constructs cloned into Flag-epitope tag vectors (G). HEK293T cells were transfected without or with HA-OTUD7B in combination with the indicated Flag-p53 constructs before subjecting cell lysates to IP with anti-Flag antibodies followed by Western blot analysis of input lysate and IP samples (H). **I.** Western blot analyses measuring OTUD7B and p53 expression in HepG2 (left) and SMMC-7721 (right) cells after fractionation of homogenates (Hm) into cytoplasmic (Cy) and nuclear (Nu) compartments. **J.** Representative confocal images showing immunofluorescence staining against OTUD7B (green), p53 (red), and DAPI (blue) in HepG2 and SMMC-7721 cells (bar = 20 µm). **K.** Association between endogenous forms of OTUD7B p53 in MHCC-97H and Huh7 cells after IP and Western blot analysis. **L.** HEK293T cells were transfected with HA-OTUD7B in combination with Flag-tagged wildtype p53 (Flag-p53-WT) or the corresponding R175H and G279E mutant constructs before subjecting cell lysates to IP with anti-Flag antibodies followed by Western blot analysis. Data information: The experiments shown were repeated three times with data shown as mean ± SD of three independent experiments. One, two, and three asterisks indicate p < 0.05, p < 0.01, and p < 0.001, respectively.

In this report, we sought to better understand the contribution of OTUD7B to liver cancer tumorigenesis. To first verify the expression status of OTUD7B in hepatocellular carcinoma, we performed immunohistochemical (IHC) staining against a commercial tissue microarray containing 15 pairs of liver cancer and normal adjacent tissues. Consistent with the literature showing downregulation in HCC, we observed that staining for OTUD7B was frequently reduced in cancer compared to normal liver tissues (Figure 1A). Thereafter, to identify tools for further analysis, we screened a small panel of HCC cell lines using Western blotting and qPCR, respectively. Similar to tissues, the protein and mRNA levels of OTUD7B in three liver cancer cell lines (HepG2, SMMC-7721 and SK-Hep-1) were reduced relative to the normal liver cell line THLE-2 (Figure 1B-C).

Since protein-protein interactions are key to understanding molecular mechanisms, we next identified potential binding partners of OTUD7B in HCC. Using an unbiased mass spectrographic approach, immunoprecipitation (IP) of OTUD7B from HEK293T cells recovered OTUD7B along with the identification of p53 as a potential interacting protein (Figure 1D). Further IP experiments showed that both Flag-OTUD7B and Flag-p53 were able to co-precipitate the endogenous forms of p53 and OTUD7B in 293T cells, respectively (Figure 1E). Moreover, interactions between the endogenous OTUD7B and p53 proteins were demonstrable in HepG2 cells (Figure 1F). Further mapping experiments were then conducted using a range of p53 deletion constructs (Figure 1G) towards defining the regions of interaction between OTUD7B and p53. We found p53 constructs individually lacking the TAD, PRD, DBD and TD + CTD domains retained the ability to interact with HA-OTUD7B (Figure 1H, left) while constructs lacking both TAD and the DBD domains lost OTUD7B binding function (Figure 1H, right). Thus, OTUD7B physically associates with p53 through dual interfaces within the p53 TAD (1-60) and DBD (98-292) regions.

Additional characterization using nuclear-cytoplasmic separation showed a biased distribution of OTUD7B within the cytoplasm of HCC cells while p53 occurred both in the cytoplasm and nucleus (Figure 1I). Nonetheless, confocal microscopy of immunofluorescence staining showed colocalization between OTUD7B and p53, albeit mainly in the cytoplasm (Figure 1J). Furthermore, given both HepG2 and SMMC-7721 cells are known to express wildtype (WT) p53, it was important to determine if p53 mutations which are prevalent in HCC affect OTUD7B-p53 interactions. IP experiments in MHCC-97H (harboring R249S *TP53*) and Huh7 (harboring Y220C *TP53*) cells confirmed interactions between endogenous OTUD7B and mutant p53 (Figure 1K). Consistently, further co-immunoprecipitation assays in 293T cells indicated WT-p53 along with p53-R175H and p53-G279E mutants were recovered with ectopic HA-tagged OTUD7B (Figure 1L). Collectively these data establish that OTUD7B interacts with both wildtype and mutant forms of p53.

### OTUD7B promotes p53 transcriptional activity

**Figure 2:**
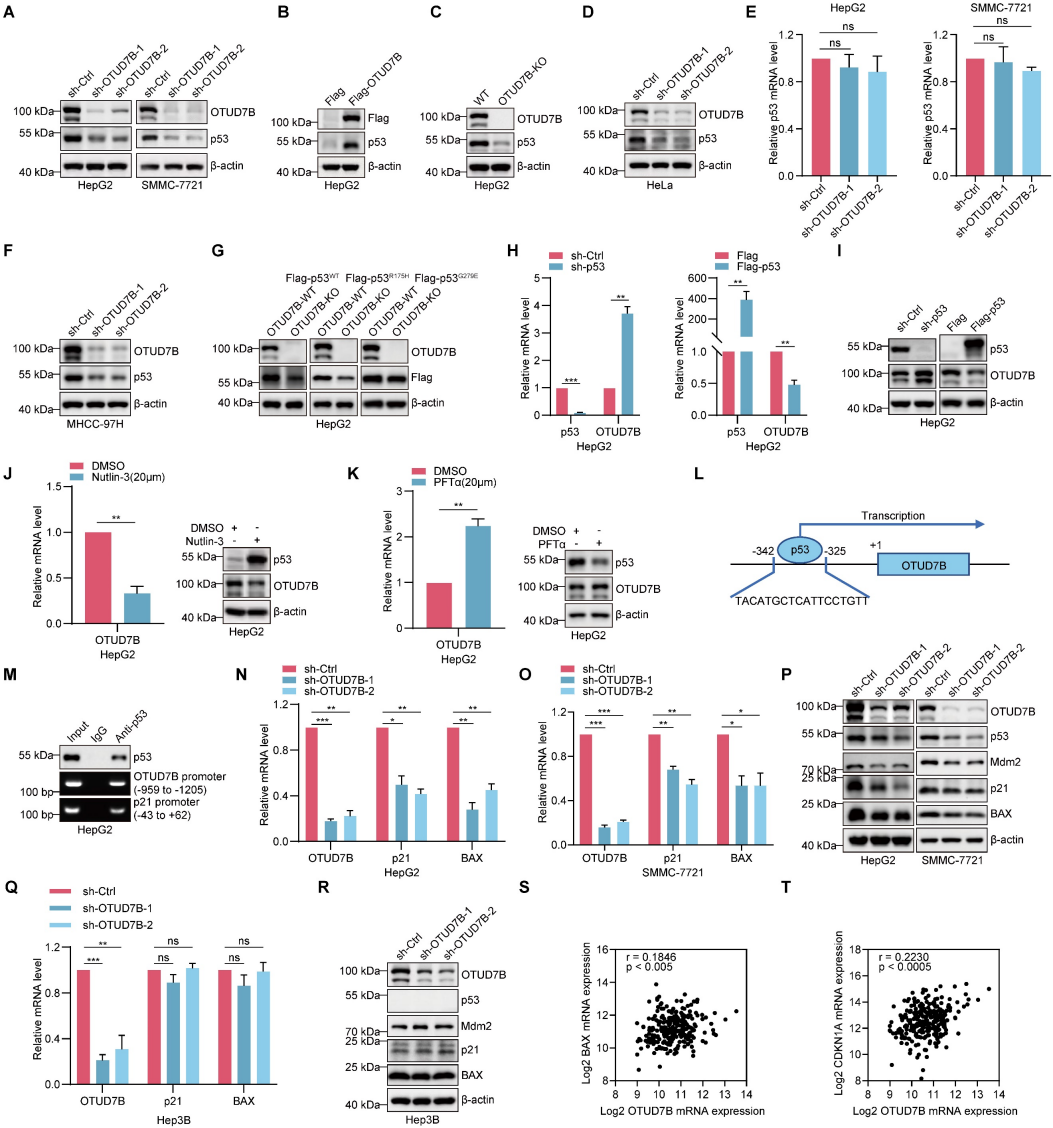
** OTUD7B enhances the expression and transcriptional activity of p53. A**. Comparative levels of p53 in HepG2 (left) and SMMC-7721 cells (right) after transduction with two independent shRNAs targeting OTUD7B (sh-OTUD7B-1 and sh-OTUD7B-2) in comparison with a control shRNA (sh-Ctrl) as determined by Western blot. **B-C.** Western blot detecting p53 levels in HepG2 cells transfected with Flag control or Flag-OTUD7B (B) or in WT and OTUD7B-knockout (KO) HepG2 cells (C). **D.** Western blot analysis of p53 levels in HeLa cells after transduction with either sh-Ctrl or independent shRNAs targeting OTUD7B. **E.** Relative p53 mRNA levels as determined by qPCR in HepG2 and SMMC-7721 cells with or without OTUD7B knockdown. **F.** Western blot analysis of p53 levels in MHCC-97H cells after transduction with either sh-Ctrl or independent shRNAs targeting OTUD7B. **G.** Western blot detecting Flag levels in HepG2 cells transfected with Flag-p53^WT^, Flag-p53^R175H^ or Flag-p53^G279E^ in WT and OTUD7B- KO HepG2 cells. **H.** Relative OTUD7B mRNA levels as determined by qPCR in HepG2 cells with or without p53 knockdown or transfected with Flag control or Flag-p53. **I.** Western blot detecting OTUD7B levels in HepG2 cells with or without p53 knockdown or transfected with Flag control or Flag-p53. **J-K.** HepG2 cells were treated with Nutlin-3 (20 μM, 24 h) (J) or PFTα (20 μM, 24 h) (K) and the levels of OTUD7B detected by qPCR and Western blot, respectively. **L-M.** Schematic showing the location of the p53 binding motif in the OTUD7B gene (L). ChIP assays against p53 or IgG control conducted in HepG2 cells with recovery of OTUD7B promoter fragments measured using PCR (M). The p21 promoter was included as a positive control for p53 binding. **N-O.** QPCR analysis showing relative mRNA levels of OTUD7B, p21 and BAX in HepG2 cells (N) or in SMMC-7721 cells (O) with or without OTUD7B knockdown. **P.** Western blot analysis of p53, Mdm2, p21, BAX levels in HepG2 and SMMC-7721 cells transduced with sh-Ctrl or two independent-shRNAs targeting OTUD7B. **Q.** Relative OTUD7B, p21 and BAX mRNA levels in p53-deficient Hep3B cells with or without knockdown of OTUD7B using qPCR. **R.** Western blotting showing p53, Mdm2, p21, BAX levels in p53-null Hep3B cells after transduction with either sh-Ctrl or two independent-shRNAs targeting OTUD7B. **S-T.** The correlation between OTUD7B and p21 (CDKN1A) and BAX transcription levels was analyzed in p53-WT samples from the Hepatocellular Carcinoma (TCGA, GDC) dataset in the cBioPortal database (Spearman correlation). Data information: The experiments shown were repeated three times with data shown as mean ± SD of three independent experiments. One, two, and three asterisks indicate p < 0.05, p < 0.01, and p < 0.001, respectively.

Given its DUB function, binding with OTUD7B suggests potential substrate interactions. Accordingly, we sought to test if p53 is a novel target of OTUD7B. Supporting this possibility, the silencing of OTUD7B expression in HCC cells using two independent shRNAs resulted in the downregulation of p53 protein levels (Figure 2A) whereas the ectopic expression of OTUD7B increased p53 protein levels (Figure 2B). Consistently, using an alternative approach, p53 levels were similarly diminished in HepG2 cells after knockout (KO) of *OTUD7B* using CRISPR-Cas9 (Figure 2C). Interestingly, the regulatory effect of OTUD7B on p53 was not limited to HCC since, for example, the same phenomenon was observed in HeLa cervical cancer cells where silencing OTUD7B similarly decreased p53 protein levels (Figure 2D). Importantly, the transcript levels of p53 were not affected after silencing OTUD7B expression in HCC (Figure 2E), indicating that OTUD7B likely regulates p53 through post-translational mechanisms.

Following on from the above findings, it remained important to determine if OTUD7B also regulates the levels of mutant p53 proteins. Silencing of OTUD7B expression in p53-mutant bearing MHCC-97H cells using independent shRNAs resulted in the reductions in p53 protein levels (Figure 2F). Moreover, after expressing Flag-epitope tagged p53-WT, p53-R175H or Flag-p53-G279E proteins in OTUD7B-KO HepG2 cell lines, we observed that the levels of mutant p53 proteins were downregulated to a similar extent to wildtype p53 (Figure 2G). Thus, OTUD7B maintains its ability to regulate p53 mutant levels. Conversely, to ascertain whether OTUD7B itself was subject to p53 regulation, we examined the impact of manipulating p53 levels in HCC cells.

Notably, knockdown of p53 led to the upregulation of OTUD7B at both transcript and protein levels, whereas overexpression of p53 resulted in OTUD7B downregulation (Figure 2H-I). Further pharmacological interventions provided alternative evidence that OTUD7B is subject to transcriptional regulation by p53. Treatment of HepG2 cells with either nutlin-3 and pifithrin-α hydrobromide (PFTα) to stabilize or block p53 expression and activity, caused decreased and increased OTUD7B mRNA and protein levels, respectively (Figure 2J-K). Together these findings propose regulatory feedback between OTUD7B and p53. Suggesting regulation at the transcriptional level, interrogating the *OTUD7B* promoter region using the JASPAR database (https://jaspar.genereg.net/) identified potential p53 binding sites including one proximal to the transcriptional start site (Figure 2L). Indeed, ChIP assays showed direct binding of p53 to the -325 to 342 binding motif in the *OTUD7B* promoter (Figure 2M), supporting a direct role for p53 as a negative regulator of OTUD7B transcription.

Given the plethora of transcriptional targets of p53 38, diminishing its expression would be expected to disrupt the p53 transcriptional program in HCC. Indeed, knockdown of OTUD7B resulted in the downregulation of the key p53 target genes p21 and BAX at both the mRNA and protein levels (Figure 2N-P). Interestingly, in Hep3B cells which are known to lack p53 expression, OTUD7B knockdown failed to affect p21 and BAX levels (Figure 2P-Q). We further considered if OTUD7B also influences Mdm2 levels, namely the main E3 ubiquitin ligase that regulates p53 which itself is regulated by negative feedback involving p53 39. Notably, the knockdown of OTUD7B in HepG2 and SMMC-7721 cells led to a decrease in Mdm2 expression levels (Figure 2P), while Mdm2 levels remained unchanged in p53-null Hep3B cells (Figure 2R). Since the aforementioned IP assays showed that OTUD7B does not directly bind with Mdm2 (Figure 1F), these data suggest that the effects on Mdm2 following OTUD7B knockdown largely depend upon changes in p53.

Lastly, based on these findings we considered evidence for OTUD7B regulation of p53 target genes in *ex vivo* HCC tissues from patients. Analysis of the wildtype *TP53* cases of hepatocellular carcinoma from the TCGA GDC dataset showed a significant positive correlation between OTUD7B and the transcriptional levels of p21 (CDKN1A) and BAX in HCC (Figure 2S-T). These results suggest that OTUD7B plays an important role in regulating the transcriptional activity of p53 and expression of its downstream genes *in vivo*.

### OTUD7B directly deubiquitinates p53 to enhance its protein stability

**Figure 3:**
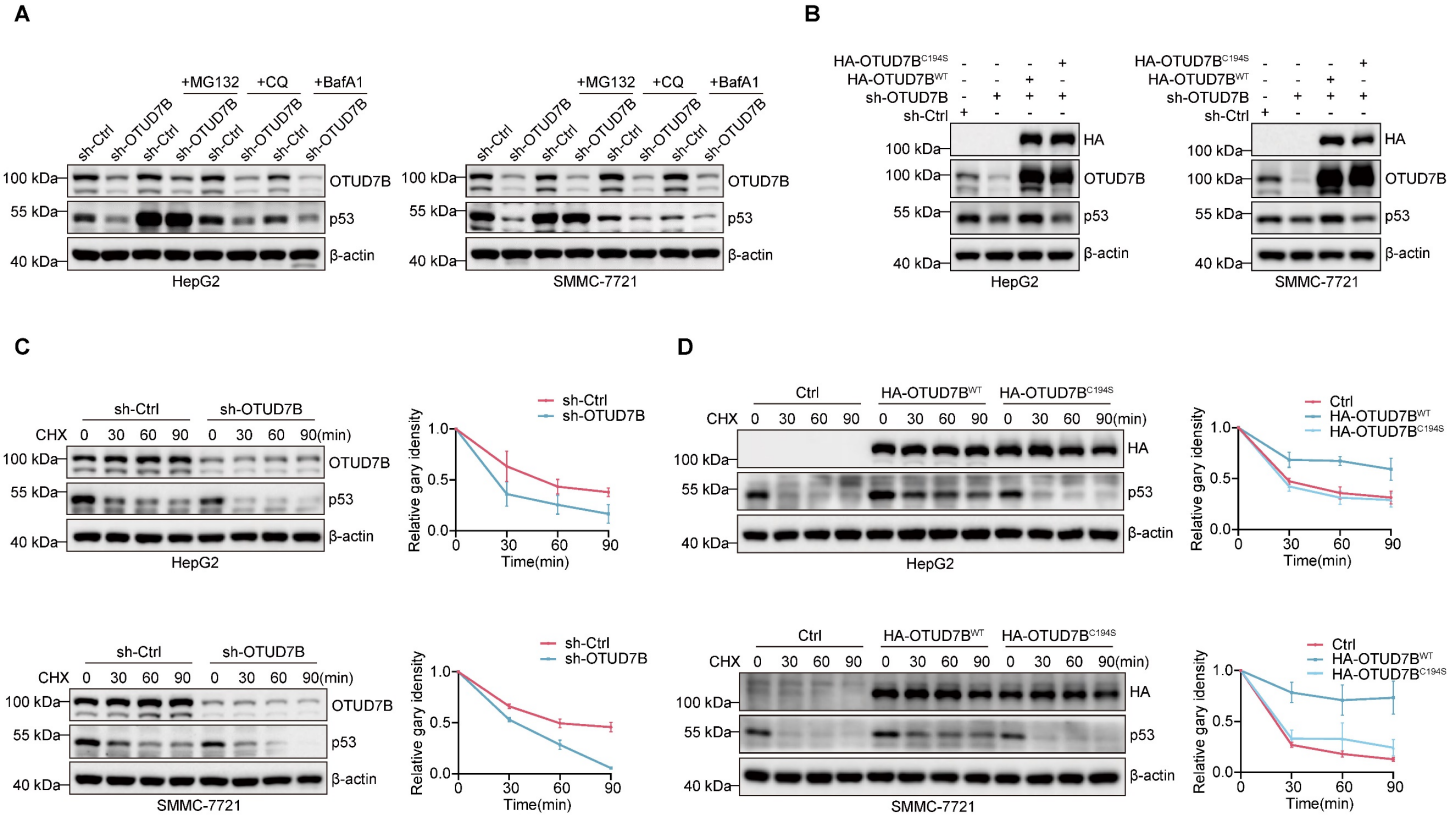
** OTUD7B enhances p53 protein stability. A.** Western blot detection of OTUD7B and p53 expression in HepG2 (left) and SMMC-7721 (right) cells transduced with sh-ctrl or sh-OTUD7B after treatment with or without MG132 (10 μM), CQ (50 μM), and BafA1 (0.2 μM) for 6 h. Blotting against b-actin was used throughout as a loading control. **B.** HepG2 (left) or SMMC-7721 (right) cells transduced with sh-Ctrl or sh-OTUD7B in combination with HA-OTUD7B-WT or HA-OTUD7B-C194S were subjected to Western blotting to measure OTUD7B, HA-OTUD7B and p53 expression. **C.** Protein stability measurements of OTUD7B and p53 in HepG2 (upper) and SMMC-7721 (lower) cells transduced with sh-Ctrl or sh-OTUD7B using 20 μg/mL CHX treatments for the indicated times. Representative Western blotting (left panels) and densitometric quantitation (right panels). **D.** Protein stability measurements of p53 determined as per (C) in HepG2 (upper) and SMMC-7721 (lower) cells after transduction with a control vector, or vectors containing HA-OTUD7B-WT or HA-OTUD7B-C194S mutant constructs. Representative Western blotting (left panels) and densitometric quantitation (right panels). Data information: The experiments shown were repeated three times with data shown as mean ± SD of three independent experiments.

We next returned to examine the posttranslational mechanism underlying OTUD7B regulation of p53 levels. Since protein degradation primarily occurs via either the UPS and/or the autophagy-lysosome pathway (ALP) 40, we implemented inhibitors of the proteasome (MG132) and autophagy-lysosome pathways (chloroquine; CQ and bafilomycin A1; BafA1) respectively. Instructively, only MG132 but not CQ or BafA1 treatment served to stabilize p53 levels in HepG2 and SMMC-7721 cells subject to OTUD7B knockdown (Figure 3A), indicative that OTUD7B regulates p53 protein levels through the ubiquitin-proteasome pathway.

The preceding findings prompted us to prepare a C194S mutant of OTUD7B (OTUD7B^C194S^) lacking DUB function to examine the hypothesis that OTUD7B DUB activity is required for regulation of p53. Comparing the effects of overexpression of OTUD7B^WT^ and OTUD7B^C194S^ in cells subject to OTUD7B knockdown showed that only wildtype OTUD7B was capable of restoring p53 protein levels (Figure 3B), supporting the conclusion that OTUD7B DUB activity is required for p53 regulation. Accordingly, the results of cycloheximide (CHX) chase assays showed that the half-life of p53 was reduced in both HepG2 and SMMC-7721 cells following the knockdown of OTUD7B (Figure 3C). Consistently, overexpression of OTUD7B^WT^ but not OTUD7B^C194S^ extended p53 protein stability (Figure 3D). These results indicate that OTUD7B regulates p53 via effects on protein stability, acting through the ubiquitin-proteasome pathway.

**Figure 4:**
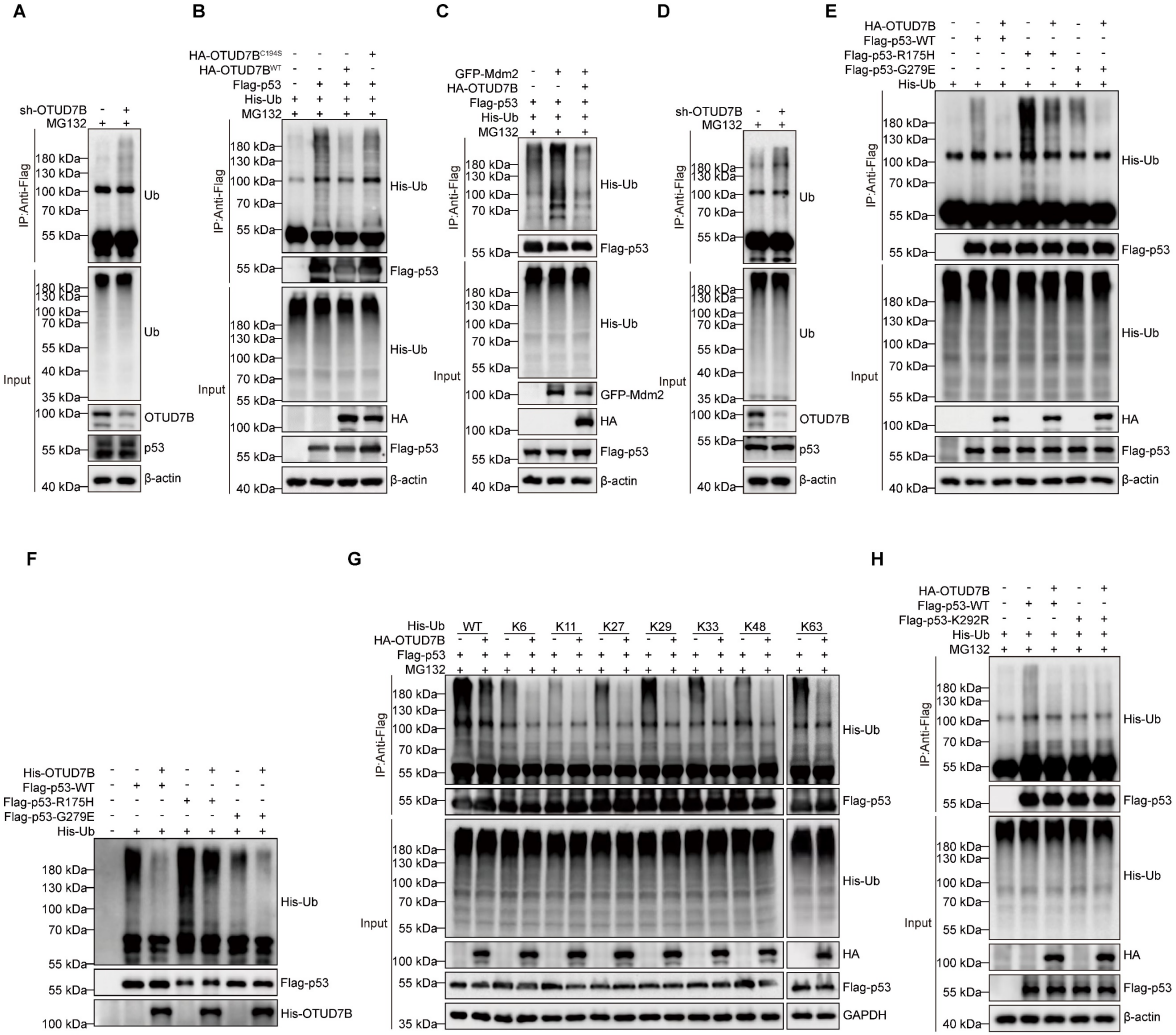
** OTUD7B functions to deubiquitylate p53. A.** Proteasomal activity was inhibited in HepG2 cells by MG132 (10 μM) treatment for 6 h after transduction with sh-Ctrl or sh-OTUD7B followed by cell lysis and IP with anti-p53 antibodies. Polyubiquitinated p53 levels were then detected by Western blotting against ubiquitin. **B.** Determination of polyubiquitylated levels of p53 in HEK293T cells expressing His-Ub in combination with or without Flag-p53 and co-expression of either HA-OTUD7B-WT or HA-OTUD7B-C194S constructs. Flag-p53 was immunoprecipitated after 6 h MG132 (10 μM) treatment and the incorporation of His-Ub assessed using anti-His Western blotting. **C.** The assay measuring polyubiquitylated Flag-p53 levels from (B) was repeated in HEK293T cells co-expressing GFP-Mdm2 alone or in combination with HA-OTUD7B. **D.** Proteasomal activity was inhibited in MHCC-97H cells by MG132 (10 μM) treatment for 6 h after transduction with sh-Ctrl or sh-OTUD7B followed by cell lysis and IP with anti-p53 antibodies. Polyubiquitinated p53 levels were then detected by Western blotting against ubiquitin. **E.** The assay from (B) was repeated to compare the effects of HA-OTUD7B overexpression on polyubiquitylation levels in wildtype or mutant (R175H or G279E) Flag-p53. **F.** *In vitro* deubiquitination assay of Ub-linked ubiquitinated p53-WT, p53-R175H, or p53-G279E enriched in recombinant OTUD7B protein and HEK293T cell extracts. The mixture was incubated at 30 °C for 4 h and then analyzed by Western blotting. **G.** The assay from (B) was repeated to compare the effects of HA-OTUD7B overexpression on the incorporation of different lysine mutants of His-Ub (His-WT, K6, K11, K27, K29, K33, K48 and K63) into Flag-p53. **H.** The assay from (B) was repeated to compare the effects of HA-OTUD7B overexpression on polyubiquitylation levels in wildtype or mutant (K292R) Flag-p53. **I.** The assay from (B) was repeated to compare the effects of HA-OTUD7B overexpression on the incorporation of different lysine mutants of His-Ub (His-WT, K6, K11, K27, K29, K33, K48 and K63) into Flag-p53.Data information: Data shown are representative of three independent experiments.

The requirement of OTUD7B DUB activity in the above experiments further implied that OTUD7B functions to regulate p53 polyubiquitination. To test this idea, we examined the relative polyubiquitination levels in p53 after various manipulations of OTUD7B. First, we observed relatively more polyubiquitinated signals in endogenous p53 immunoprecipitated from HepG2 cells following knockdown of OTUD7B (Figure 4A). Second, the incorporation of His-Ub into ectopically expressed Flag-p53 was diminished in HEK293T cells co-expressing HA-OTUD7B^WT^ but not with the OTUD7B^C194S^ mutant construct (Figure 4B). Together this indicates that OTUD7B deubiquitinates p53 to reduce its overall polyubiquitination levels. On this basis we further analyzed how OTUD7B expression affects the actions of Mdm2 that marks p53 for proteasomal degradation 41. After co-expressing Flag-p53 and GFP-Mdm2 with or without HA-OTUD7B, we found that OTUD7B removed polyubiquitin modifications in p53 catalyzed by Mdm2 (Figure 4C). Furthermore, given the observation that OTUD7B binds mutant forms of p53, it was relevant to examine the capability of OTUD7B to deubiquitinate mutant p53. Assessment of endogenous mutant p53 expressed by MHCC-97H cells showed relatively more p53-associated polyubiquitination signals after knockdown of OTUD7B (Figure 4D). Furthermore, comparing HEK293T cells co-transfected with Flag-p53-WT, Flag-p53-R175H, or Flag-p53-G279E constructs in the presence or absence of HA-OTUD7B revealed that OTUD7B significantly reduced the levels of polyubiquitination in wildtype and mutant p53 (Figure 4E). Lastly, the results of *in vitro* de-ubiquitination assays showed that the presence of recombinant OTUD7B purified from *E. coli* was sufficient to significantly reduce ubiquitination levels in wildtype and p53 mutants (Figure 4F). Together these findings establish that OTUD7B functions to de-ubiquitinate wildtype and mutant forms of p53.

Finally, to ascertain the type of ubiquitin modification present in p53 removed by OTUD7B, we repeated the ubiquitination assay after transfecting a series of ubiquitin mutants (K6, K11, K27, K29, K33, K48 and K63) where all but one lysine had not been mutated (Figure 4G). We found that the OTUD7B deubiquitinating enzyme not only effectively removes the traditional K48 and K63 ubiquitin tags in p53 associated with protein degradation but was also capable of removing non-canonical ubiquitin modifications, such as those at K6, K11, K27, K29, and K33. Further, to define ubiquitination site(s) in p53 recognized by OTUB7B, we interrogated p53 immunoprecipitated from parental HepG2 and OTUD7B-KO cells using mass spectrographic analysis. Comparative analysis identified the K292 site as the major ubiquitination site accumulating in p53 from HepG2-OTUD7B-KO cells relative to normal HepG2 cells (Figure S1A). Consistently, we found a K292R p53 mutant protein was ubiquitinated at lower levels than wildtype p53, and its ubiquitination levels were unaffected by co-transfection with OTUD7B (Figure 4H).

Collectively these experiments reveal the broad role of OTUD7B in regulating the stability and activity of p53, acting to directly deubiquitinate p53.

### P53 protein levels positively correlate with OTUD7B in HCC

**Figure 5:**
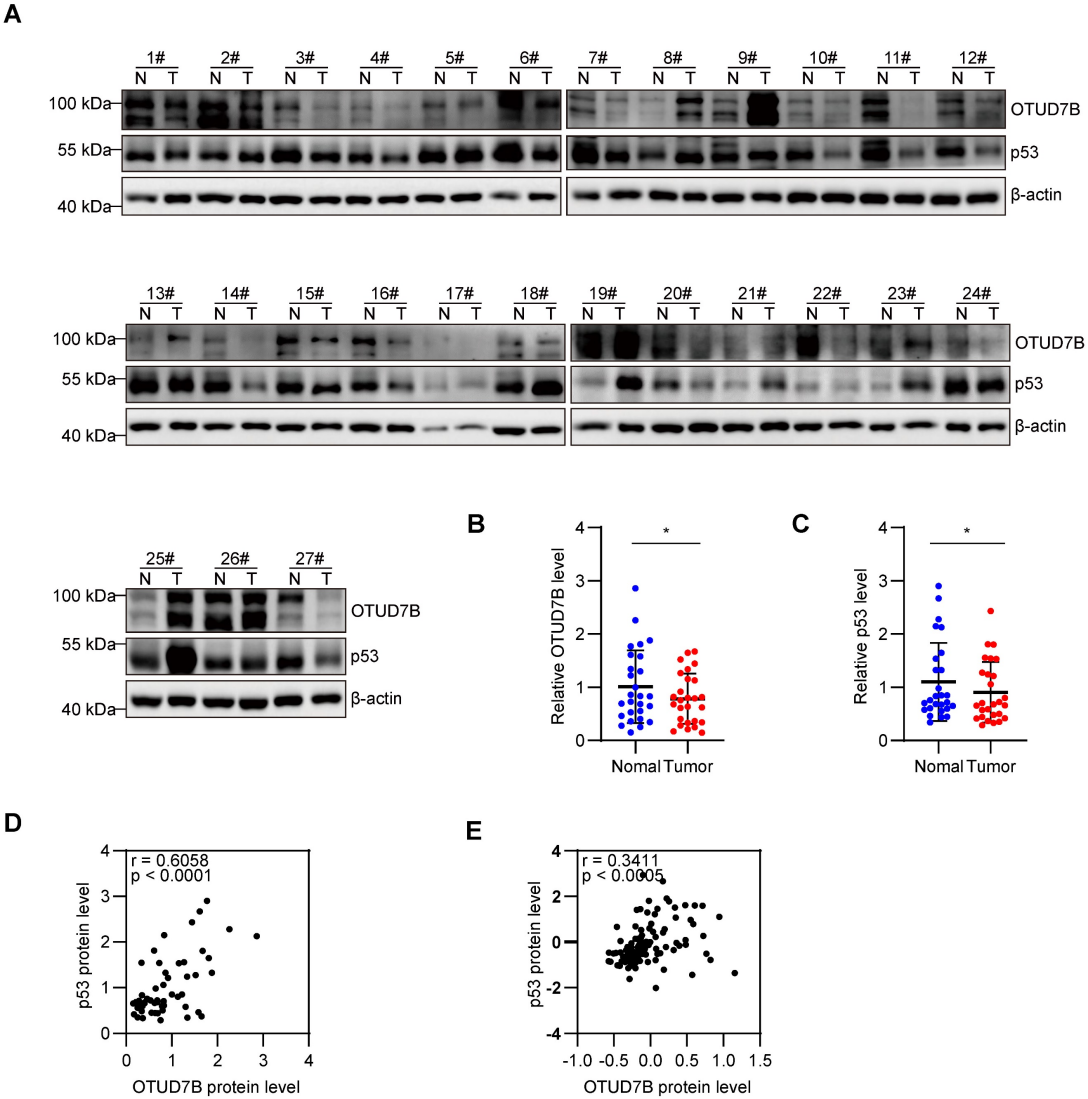
** OTUD7B protein levels positively correlate with p53 in HCC tissues. A-D.** Western blot detection of OTUD7B and p53 in 27 matched pairs of HCC and normal adjacent tissues (A). Relative OTUD7B (B) and p53 (C) protein levels in tissues from (A) quantitated using densitometry along with Pearson correlation between OTUD7B and p53 in cases of HCC (D). **E.** Pearson correlation analysis of OTUD7B and p53 protein expression in the HCC dataset, representing all 56 patients from the CPTAC using the cProSite tool. Data information: Data shown as mean ± SD with one asterisk indicating p < 0.05.

To further evaluate correlations between OTUD7B and p53 expression in liver cancer, protein extracts from 27 pairs of fresh liver vs adjacent non-cancerous tissues were subject to Western blotting against OTUD7B and p53 (Figure 5A). Overall, there were significant decreases in the levels of both OTUD7B and p53 in cancerous versus normal tissues (Figure 5B-C) with each marker being positively correlated in liver cancer tissues (Figure 5D). Consistently, utilizing the Clinical Proteomic Tumor Analysis Consortium (CPTAC) database to analyze the protein expression in liver cancer showed a significant positive correlation between OTUD7B with p53 (Figure 5E).

### OTUD7B regulates hepatocellular carcinoma cell tumorigenicity via effects on p53

**Figure 6:**
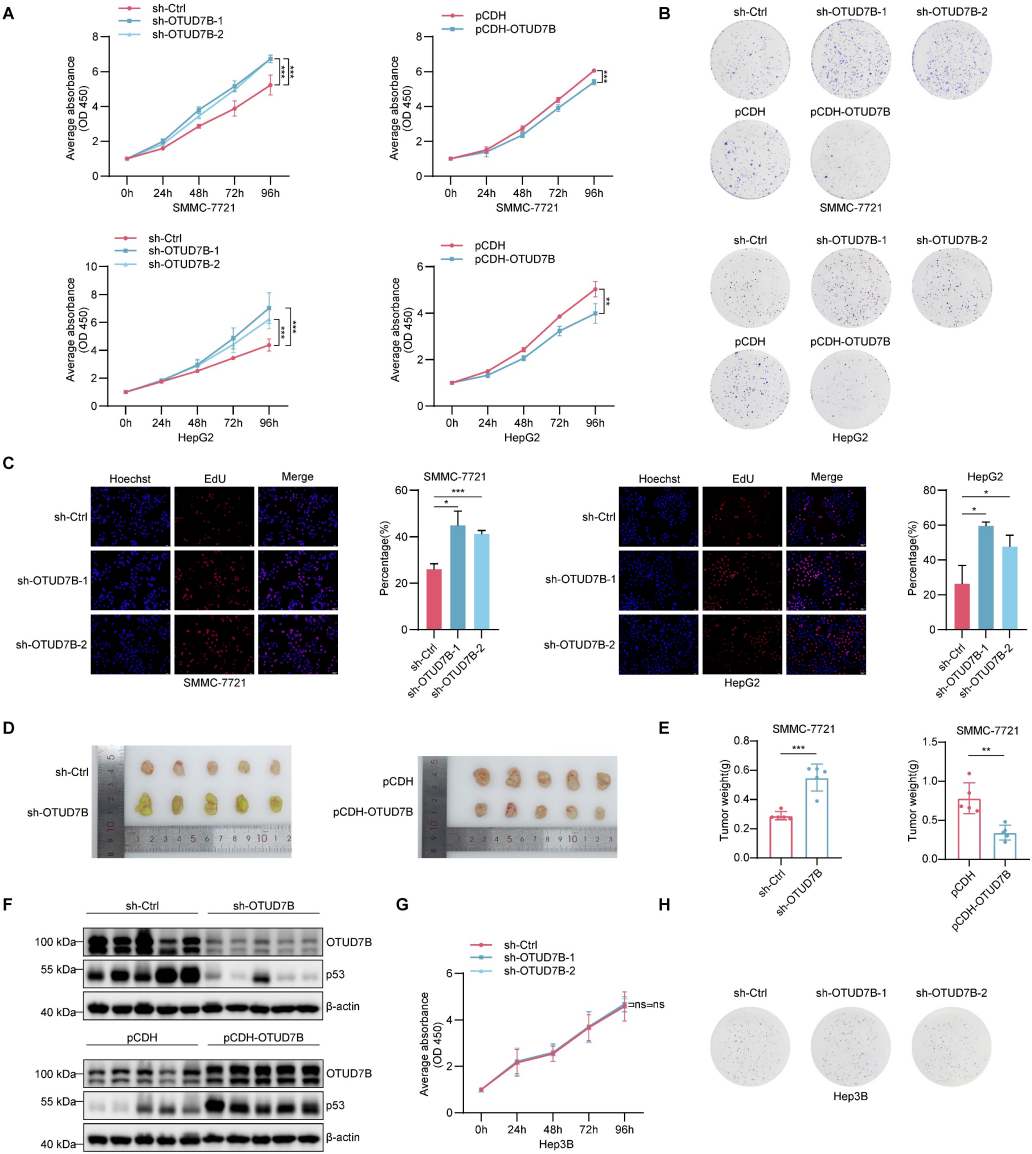
** OTUD7B regulates HCC cell proliferation *in vitro* and *in vivo*. A.** Cell proliferation of SMMC-7721 (top) and HepG2 (bottom) cells measured over 96 h using the CCK-8 assay after knockdown (left) or overexpression (right) of OTUD7B using transduction with shRNAs or pCDH vector-based constructs, respectively. **B.** Clonogenic growth of SMMC-7721 and HepG2 cells treated as per (A) assessed after 2 weeks of culture. **C.** DNA synthesis in SMMC-7721 and HepG2 cells following knockdown of OTUD7B as per (A) determined using EdU incorporation over 4 h. Representative epifluorescence images (left) and comparative DNA synthesis rates determined using image analysis (right). **D-F.** *In vivo* growth of HCC cells after knockdown or overexpression of OTUD7B as per (A). After injecting 5 x10^6^ SMMC-7721 cells into the right flanks of nude mice (n = 5 mice/group), xenografts were excised after four weeks and photographed (D) and weighed (E). Xenografts were homogenized and the altered levels of OTUD7B and p53 proteins verified by Western blotting with the indicated antibodies. **G-H.** p53-deficient Hep3B cells were subject to cell proliferation (G) and clonogenic growth (H) assays as per (A-B) after transduction with either control shRNA or two independent OTUD7B-shRNAs. Data information: *In vitro* data are representative of three experiments with all data shown as mean ± SD. One, two, and three asterisks indicate p < 0.05, p < 0.01, and p < 0.001, respectively.

Previous research involving hepatocellular carcinoma supports the idea that OTUD7B acts as a tumor suppressor 24, 42 although its connection with p53 appears novel to our best knowledge. We therefore investigated the effect of OTUD7B on tumorigenic phenotypes in HCC and its relationship with the regulation of p53. Accordingly, we first examined the effects of manipulating OTUD7B levels on cell proliferation in representative HCC cells expressing wildtype p53. The results of cell counting kit-8 (CCK-8) and colony formation assays showed that OTUD7B knockdown resulted in increased cell growth and clonogenic capability while ectopic OTUD7B overexpression retarded cell growth in SMMC-7221 and HepG2 cells (Figure 6A-B). Consistent with the increased cell proliferative activity, the incorporation rates of 5-Ethynyl-2'-deoxyuridine (EdU) were significantly increased in OTUD7B knockdown cells (Figure 6C). Importantly, the proliferative changes observed *in vitro* were reflected *in vivo* using cell line*-*derived tumor xenograft (CDX) models in nude mice. Here, OTUD7B knockdown produced significantly larger xenografts while those tumors established after OTUD7B overexpression were smaller than the controls (Figure 6D-F).

We then looked further at the relationship between OTUD7B expression, *TP53* mutation and HCC growth. We repeated the OTUD7B knockdown experiments in MHCC-97H cells which bear the gain of function (GOF) R249S "hotspot" *TP53* mutation. In contrast to wildtype *TP53* cells, silencing OTUD7B in MHCC-97H cells diminished cell growth and colony formation (Figure S2A-B), suggesting OTUD7B regulation of mutant p53 impinges on p53 GOF activity. Moreover, we compared the growth potential of different p53-null HCC lines after OTUD7B knockdown including Hep3B cells with endogenous loss of p53 or HepG2 and SMMC-7721 rendered p53 null using CRISPR-Cas9. This analysis revealed that the growth inhibitory effects of OTUD7B knockdown were absent in HepG3 cells (Figure 6H-I) and lost in p53-KO HepG2 and SMMC-7721 cells (Figure S2C-F). Together these findings suggest that the phenotypic changes in HCC cells elicited by OTUD7B rely on the downstream actions of p53.

### OTUD7B promotes apoptosis of hepatocellular carcinoma cells through p53

**Figure 7:**
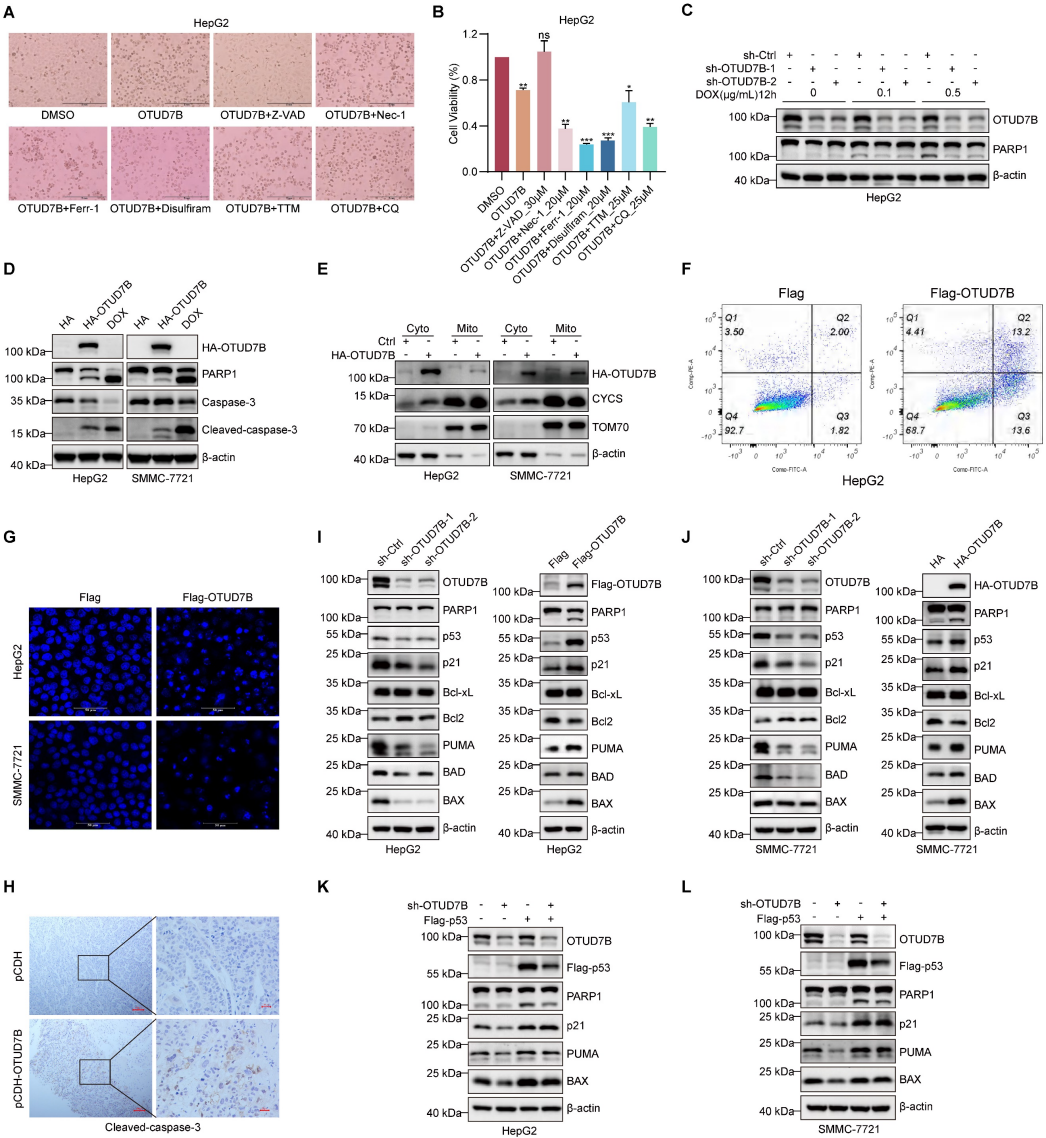
** OTUD7B induces apoptosis in HCC cells. A-B.** Representative phase-contrast images of HepG2 cells without or with OTUD7B overexpression treated with DMSO carrier compared with inhibitors of apoptosis (30 μM Z-VAD), necrosis (20 μM Nec-1), ferroptosis (20 μM Ferr-1), pyroptosis (20 μM Disulfiram), cuproptosis (25 μM TTM), and autophagy (25 μM CQ), respectively (A). Cell viability measurements determined from CCK-8 assays (B). **C.** HepG2 cells transduced with sh-Ctrl or two independent shRNAs targeting OTUD7B were treated with doxorubicin (0, 0.1, 0.5 μg/mL) for 12 h before immunoblotting cell lysates to measure PARP cleavage and to verify OTUD7B knockdown. **D-E.** The apoptotic marker proteins PARP and caspase-3 were detected in HepG2 and SMMC-7721 cells by immunoblotting after transfection with either HA control or HA-OTUD7B. Doxorubicin (2.5 μg/mL) treatment served as a positive control (D). Alternatively, the cells from (D) were fractionated and the levels of CYCS release from mitochondrial (Mito) to cytoplasmic (Cyto) compartments compared (E). b-actin and TOM70 served as cytoplasmic and mitochondrial markers, respectively. **F.** Flow cytometric plots comparing annexin V-FITC/PI double staining in HepG2 cells after transfection with Flag control or Flag-OTUD7B vectors for 24 h. **G**. Representative epifluorescence images of DAPI staining visualizing cell nuclei in HepG2 and SMMC-7721 cells after transfection with Flag control or Flag-OTUD7B vectors for 24 h (bar = 20 µm). **H.** Micrographs of IHC staining against cleaved caspase-3 in sections of SMMC-7721 xenografts comparing control and OTUD7B overexpressing tissues. **I-J.** Immunoblotting analyses against OTUD7B, p53 and the indicated apoptotic regulatory proteins in HepG2 (I) and SMMC-7721 (J) cells without or with OTUD7B knockdown (left panels) or without or with OTUD7B overexpression (right panels). **K-L.** Immunoblotting analyses against OTUD7B, Flag-p53 (anti-Flag), PARP1, p21, PUMA and BAX in HepG2 (K) and SMMC-7721 (L) cells transduced with or without sh-OTUD7B in combination with or without Flag-p53. Data information: The experiments were repeated three times and data shown as the mean ± SD. One, two, and three asterisks indicate p < 0.05, p < 0.01, and p < 0.001, respectively.

The above results are consistent with a tumor-suppressor role for OTUD7B in liver cancer. But exactly how does OTUD7B inhibit tumorigenicity in liver cancer cells? Along with cell cycle arrest 43, p53 is known to play key roles in regulating various types of cell death, including apoptosis 44, autophagic cell death 45, cuproptosis 46, ferroptosis 47, and necroptosis 48. Moreover, previous research reported that OTUD7B promotes apoptosis in liver cancer cells by negatively regulating NF-κB 24. Here we casually observed that increased cell death was evident upon overexpression of OTUD7B in HepG2 cells (Figure 7A). To clarify which mode(s) of cell death were invoked by OTUD7B, we assessed the ability of selective cell death inhibitors to antagonize the effects of ectopic OTUD7B expression. Among those tested including the apoptosis inhibitor Z-VAD-FMK (Z-VAD), the programmed necrosis inhibitor necrostatin-1 (Nec-1), the ferroptosis inhibitor ferrostatin-1 (Ferr-1), the necroptosis inhibitor Disulfiram, the cuproptosis inhibitor tetrathiomolybdate (TTM), and the autophagy inhibitor CQ; however, cell viability reductions caused by OTUD7B overexpression were only restored by the addition of the apoptosis inhibitor Z-VAD (Figure 7A-B). Conversely, the amount of cleaved PARP, a marker of apoptosis, was reduced in cells subject to DNA damage with doxorubicin when OTUD7B levels were reduced by shRNA-mediated knockdown (Figure 7C). Moreover, consistent with the idea that OTUD7B promotes apoptosis, the overexpression of OTUD7B resulted in higher levels of cleaved PARP1 and caspase-3 relative to control conditions (Figure 7D). And consistent with OTUD7B-induced apoptosis mainly proceeding through the mitochondrial-dependent pathway, cytochrome c (CYCS) release from mitochondria to the cytoplasm was evident in cells following the overexpression of OTUD7B (Figure 7E). Other phenotypic features consistent with apoptosis observed upon overexpressing OTUD7B in HCC cells were the condensation and fragmentation of nuclear chromatin 49 as detected by flow cytometry and confocal microscopy, respectively (Figure 7F-G), while examination of cleaved caspase 3 staining in HCC xenografts showed that OTUD7B overexpression also increased HCC cell apoptosis rates *in vivo* (Figure 7H). Thus, OTUD7B plays a definitive role in promoting apoptosis in liver cancer cells.

Cell apoptosis can be regulated by p53 both transcriptionally and non-transcriptionally where notably p53 can directly interact with members of the Bcl-2 family 50, 51. Given our preceding data linking the effects of OTUD7B with p53, it was relevant to explore downstream factors associated with p53-dependent apoptosis. Using Western blotting against HepG2 and SMMC-7721 cells subject to both OTUD7B knockdown and overexpression (Figure 7I-J), respectively, we observed that knockdown of OTUD7B led to the upregulation of the anti-apoptotic protein Bcl-2 but not Bcl-xL, while the pro-apoptotic proteins BAX, BAD, and PUMA were downregulated along with p21. Countering these findings, OTUD7B overexpression resulted in decreased Bcl-2 expression while BAX, BAD, and PUMA were increased together with p21. Moreover, changes in BAX, PUMA and p21 in HepG2 and SMMC-7721 cells were lost after p53 knockout (Figure S3A-B). Furthermore, showing the importance of p53 expression in this setting, restoring p53 expression in HepG2 and SMMC-7721 cells with OTUD7B knockdown led to more PARP1 cleavage and restored expression levels of p21, PUMA, and BAX (Figure 7K-L). The above results establish that OTUD7B is involved in regulating apoptosis in liver cancer cells by modulating the expression levels of p53.

## Discussion

The activation of p53 is crucial for the maintenance of normal cells, serving to protect from damage events that could otherwise initiate transformation. However, approximately half of all cancers exhibit somatic alterations within the *TP53* gene including mutations providing either loss of its tumor-suppressive function (LOF) or even acquiring new oncogenic functions, known as GOF mutations 52, 53. In turn, such mutations disavow p53's normal functions, promoting tumorigenesis through a range of processes including effects on cell proliferation, metastasis, genomic instability, differentiation and stemness, metabolic reprogramming, tumor microenvironmental effects, immune responses, and resistance to cancer therapies 54, 55. This critical role has promulgated p53 as a priority therapeutic target although significant challenges exist in developing targeted therapeutics, in large part due to the absence of suitable 'drug pockets' in p53 together with the lack of established mechanisms for protein reactivation 56. The prominent role of the ubiquitin-proteasome pathway in protein regulation has also been pursued, with the E3 ubiquitin ligase Mdm2 being identified as the primary negative regulator of p53 which can decrease p53 protein levels and suppress its tumor-suppressive function 57. Notably, Mdm2 itself functions as an oncogene, with for example, its overexpression in many sarcomas inactivating p53 in an alternate manner 58. In response, a range of different drugs targeting Mdm2 developed to counteract its regulation of p53 and other key targets 59. On the other hand, DUB enzymes prevent target protein degradation by counteracting the degradation signals from ubiquitin ligases. The enzymes which deubiquitinate p53 are less well-studied with this report revealing a regulatory nexus between p53 and the OTUD7B in liver cancer.

Studies focusing on cancer have generally concluded that OTUD7B is typically expressed at high levels in tumor cells, often as a result of genomic amplification 18, 20, where it variously functions to promote tumorigenesis. However, the case of HCC is exceptional given contrasting observations of lowered OTUD7B expression which is associated with disease recurrence and reduced overall survival 24, 25. Here using several methodical approaches, we confirmed the differential reduction of OTUD7B in HCC compared to normal liver tissue, reinforcing its unique role in the context of liver cancer. Nonetheless, it remains unclear why OTUD7B expression decreases progressively according to HCC stage 24, 25. Others have shown retinoic acid (RA) treatment increases OTUD7B levels in HCC 24 while we observed that OTUD7B was subject to transcriptional repression by p53: the latter finding further suggests that p53 establishes negative feedback towards tuning the cellular levels of OTUD7B. It is further noteworthy to consider how the wiring of the OTUD7B-p53 relationship may dovetail with the well-known Mdm2-p53 regulatory circuit. We observed that OTUD7B was effective in removing different classes of polyubiquitin chains from p53 including those added by Mdm2, proposing competition for p53 as substrate. A contrasting relationship also exists where Mdm2 and OTUD7B expressions are promoted or repressed, respectively, through p53-dependent transcriptional mechanisms. Based on our observations, we speculate that the relatively low levels of p53 maintained by Mdm2 60 provide a permissive state for OTUD7B transcription at a subdued level, with OTUD7B actions providing an extra layer of control over p53. However, further studies are needed to reveal the complexity of the mechanisms involved.

Our results clearly show that both wildtype and common p53 mutant forms of p53 are subject to positive regulation by OTUD7B DUB activity. To our best knowledge, no previous reports have identified p53 as a target of OTUD7B, although the related OTUD3 protein, which like OTUD7B displays either tumor suppressor or oncogenic functions according to tumor type, was previously reported to target p53 in breast cancer 7. Moreover, we detected positive correlations between OTUD7B and p53 levels in clinical HCC samples which likely reflect that the regulatory relationship identified in our study is relevant to patients. Nevertheless, the limitations of our analysis such as small sample size must be considered along with the need to better analyze the relationship between OTUD7B and different types of p53 mutants. In this regard, the high rates of p53 mutation in HCC and other cancer types have provided complexity to the therapeutic targeting of p53, not to mention the existence of different mutant forms. Consequently, strategies to target mutant p53 still have significant limitations and face major challenges in practice.

Major conceptual approaches for targeting mutant p53 mainly include restoring wild-type p53 function, as seen in the study by Chen *et al*., which found that arsenic trioxide (ATO) reacts with specific amino acid residues within the p53 protein by a "hidden allosteric site," thus aiding in the restoration of p53's normal structure and function 61. Other ideas proposed include inducing mutant p53 degradation or otherwise inhibiting interactions with key binding proteins or nullifying key downstream pathways engaged by p53 mutants. Our discovery that OTUD7B maintains functional interactions with different p53 mutants promotes the idea of utilizing OTUD7B as a therapeutic target. Foremost, we showed that increasing OTUD7B expression accentuated p53 expression, causing growth inhibition while its knockdown promoted HCC cell growth both *in vitro* and *in vivo*. Importantly these effects were entirely absent after manipulating OTUD7B in p53 null HCC cells, highlighting the relationship between OTUD7B and HCC cell proliferation via wildtype p53-dependent effects. On the other hand, depleting OTUD7B in HCC cells expressing the R249S p53 mutant caused growth reductions, consistent with its GOF activities reported to involve interactions with transcriptional factors such as c-Myc 62. Thus, addressing the diminished OTUD7B levels in HCC would notionally restore p53 expression and function whereas targeting OTUD7B could help eliminate the effects of p53 GOF mutants. In this regard, the context of p53 genetic background in individual tumors remains a critical consideration, for example, to define the extent of benefit for restoring the weakened expression of LOF p53 mutants. Another caveat concerns balancing the actions of OTUD7B against other protein clients. For instance, OTUD7B functions to prevent TRAF3 degradation in lung cancer cell cells, suppressing their invasiveness though the non-canonical NF-κB pathway 23. This study represents one of several reports showing both negative and positive effects of OTUD7B on tumor cell invasiveness 18, 23, 42, 63, a tumorigenic phenotype which we have not addressed here.

We further observed one of the major outcomes associated with the stabilization of p53 via enforced OTUD7B expression involved inducing HCC cell apoptosis, phenocopying the p53 response to cellular insults such as unresolved levels of DNA damage. P53 is well known for promoting apoptosis, commonly via transcriptional activation of pro-apoptotic Bcl-2 family members such as BAX, PUMA, Noxa, BAD, and Bid, while inhibiting the transcription of other related anti-apoptotic genes, including Bcl-2 and Bcl-xL 64. Here we observed that apoptosis induction resulting from ectopic OTUD7B expression was strongly associated with the p53-dependent expression of PUMA and BAX, acting to eliminate HCC cells via the mitochondrial apoptotic signaling pathway. This is entirely consistent with the respective roles of p53 and the Bcl-2 family proteins which act as central regulators of mitochondrial apoptosis 32, 65. Since many anti-cancer drugs achieve effects through inducing apoptosis, there may be further value in investigating their potential synergy with approaches to manipulate OTUD7B expression in HCC.

## Methods

### Reagents

Sources for general reagents and antibodies are shown in Table S1 and S2, respectively; oligonucleotide sequences used for shRNA, knockout and PCR experiments are stated in Table S3, S4 and S5, respectively; recombinant DNA constructs used are listed in Table S6; ChIP primer sequences are listed in Table S7.

### Cell culture

Human liver cancer cell lines HepG2, SMMC-7721, Hep3B, SK-Hep-1 and THLE-2, human cervical cancer cell line HeLa, and human embryonic kidney cell line 293T were maintained at 37 °C with 5% CO_2_ humidified atmosphere. The culture medium used was complete DMEM (Dulbecco's Modified Eagle Medium) supplemented with 10% fetal bovine serum, 1 mM sodium pyruvate and 100 mg/mL penicillin-streptomycin (Gibco). All cells were authenticated by short tandem repeat (STR) analysis and tested negative for mycoplasma contamination.

### Lentiviral infections and gene transfection

The CRISPR Cas9 gene knockout system comprising pVSVg, psPAX2 and lentiCRISPRv2 (1:2:2), the shRNA knockdown system comprising pGIPZ, GAG, and VSVG (2:2:1), or the overexpression system comprising pMD2.G, psPAX2, and pCDH (1:2:2) were used to co-transfect 293T cells using Lipofectamine 2000 (Invitrogen#11668019) at the indicated vector ratios. After 48 h, viral supernatants were harvested and filtered using a 0.45 μM filter (Millipore) and mixed at a 1:1 ratio with fresh culture medium before addition to target cells in the presence of 8 μg/mL polybrene (Sigma). After 12 h, the cells were selected with 1-5 μg/mL puromycin (Sigma). Alternatively, gene transfection with the pCMV-HA and p3 × Flag-Myc-based plasmids was conducted with Lipofectamine 2000 according to the manufacturer's recommendations.

### Cell viability and colony-formation assays

To measure cell viability, equal numbers of cells seeded into 96-well plates were subjected to the indicated treatment conditions before addition of 10 μL/well CCK-8 solution (Dojindo) followed by further incubation at 37 °C. Optical density (OD) at 450 nm was measured using a microplate reader and data normalized to the control condition. To measure colony outgrowth, 1,000 cells/well seeded into 6-well plates were cultured for two weeks, followed by washing, fixation (4% formaldehyde for 20 min at RT) and staining with 0.2% w/v crystal violet. Whole wells were imaged for analysis.

### RNA extraction and qRT-PCR

Total RNA was extracted from cells using the TRIZOL Reagent (Invitrogen) according to the manufacturer's instructions. 1 μg of total RNA was transcribed into cDNA using the PrimeScript^TM^ RT reagent Kit (TaKaRa) before performing qPCR reactions with the specified primers (Table S5) and the TB Green Premix Ex Taq II (Tli RNaseH Plus) (TaKaRa) reaction mixture.

### Western blotting

SDS Loading Buffer was added to cell or tissue homogenates to a final 1 × concentration before heat denaturation and separation by denaturing SDS-PAGE. After transfer onto PVDF membranes, the samples were blocked for 1 h with 5% skim milk and incubated with primary antibodies overnight at 4 °C before washing, incubation with species appropriate secondary antibodies for 1 h at room temperature. Thereafter, bands were visualized with a chemiluminescent substrate and images captured for later analysis. Image J software was used to quantify band densities where indicated.

### Immunoprecipitation (IP)

After harvesting, cells were resuspended at ~10^7^ cells/mL in IP buffer (20 mM Tris, 150 mM NaCl, 1.5 mM MgCl_2_.6H_2_O, 1 mM EDTA, pH 7.8, 0.5% NP-40, 5% Glycerol) containing proteinase inhibitors (2% v/v cocktail (Roche) and 1% v/v PMSF solution). After incubation for 1 h at 4 °C, the samples were clarified by centrifugation at 12,000 × g for 15 min at 4 °C. Afterwards, supernatants were incubated with protein A/G Beads precoated with antibodies (4 °C for 2 h), at 4 °C for 4 h or overnight. After thrice washing with IP buffer, the beads were eluted by heat denaturation in 2 × loading buffer before analysis.

### ChIP assays

Assays were performed according to the manufacturer's instructions using the ChIP Detection Kit (CST). Briefly, approximately ~ 4 × 10^6^ adherent cells were fixed with 1% formaldehyde at room temperature for 10 min, with the cross-linking reaction terminated by glycine (0.125 M) addition for 5 min at room temperature. After collecting the cells and centrifugation at 4 °C, cell pellets were lysed in 300 µL of SDS lysis buffer for 15 min, and chromatin sheared to 200-1000 bp using sonication. The resulting chromatin fragments were incubated overnight with an anti-p53 antibodies followed by capture of the antibody-protein-DNA complexes using protein A/G magnetic beads. The beads were sequentially washed with low salt buffer, high salt buffer, LiCl buffer, and TE buffer to reduce non-specific binding and protein-DNA complexes released with elution buffer (1% SDS and 0.1 M NaHCO3). Thereafter, cross-links were reversed by incubating the samples at 65 °C for 2 h in the presence of 5 M NaCl and proteinase K. The recovered DNA was then purified and subjected to PCR using specific primers (Table S7).

### Immunofluorescence staining

Cells grown on coverslips were fixed with 4% formaldehyde at room temperature, permeabilized with 0.1% Triton X-100 for 5 min, and then blocked with 5% BSA solution for 30 min at room temperature. Samples were then sequentially incubated with primary antibodies for 1 h at room temperature and washed before incubation with the corresponding secondary antibodies for 30 min at room temperature. Cell nuclei were counterstained with DAPI for 2 min and coverslips mounted onto slides using antifade reagent. Stained cells were visualized using epifluorescence microscopy and confocal images collected using an TCS SP8 CLM (Leica).

### Ubiquitination and de-ubiquitination assays

Cells were co-transfected for 48 h with the indicated combinations of constructs (Table S6) before treatment with 10 μM MG132 for a further 6 h. The cells were harvested and resuspended in 1 mL of dilution buffer (20 mM Tris, 150 mM NaCl, 1.5 mM MgCl2·6H2O, 1 mM EDTA, pH 7.8, 0.5% NP-40, 5% glycerol, 2% cocktail, 1% PMSF) and rotated at 4 °C for 1 h. The samples were clarified by centrifugation at 12,000 × g for 15 min at 4 °C and supernatants further incubated with M2-anti-Flag beads with rotation at 4 °C for 4 h. After washing three times, the samples were eluted by heat denaturation in 1 × Loading Buffer before Western Blot to detect ubiquitinated proteins. For de-ubiquitination assays, p53 substrate proteins were purified from HEK293T cells co-transfected with Flag-tagged p53-WT, p53-R175H, or p53-G279E in addition to His-Ub. Prior to recovery by IP with M2-anti-Flag beads, the cells were treated with 10 μM MG132 for 6 h to enhance ubiquitinated protein levels. The beads were washed with a deubiquitinating buffer (60 mM HEPES, 5 mM MgCl2, 4% glycerol, pH 7.6) three times and incubated with recombinant His-OTUD7B protein (purified from E. coli) at 30 °C for 4 h. The mixture was boiled in 2 × SDS-PAGE loading buffer and analyzed by Western blot.

### Identification of p53 ubiquitination sites

Immunoprecipitations were conducted against p53 from HepG2-WT and HepG2-OTUD7B-KO cells, followed by SDS-PAGE separation and Coomassie Blue staining. Bands corresponding to p53 bands were excised from the gel and subjected to LC-MS/MS analysis by QLBio.

### Apoptosis assay

Dual apoptosis assays based on Annexin V binding and PI permeability were conducted using the Annexin V-FITC/PI staining kit (BD, 556547) according to the manufacturer's instructions. Briefly, both adherent and floating cells in supernatants were combined, washed with precooled PBS, before resuspension in 1 × Annexin-binding buffer containing 5 μL of FITC Annexin V and 5 μL of PI. After incubating in the dark at RT (25 °C) for 15 min, the rates of cell apoptosis were determined by flow cytometry within 1 h.

### Immunohistochemistry

FFPE tissue sections including commercial tissue arrays (HLiv030PG05-1; OUTDO BIOTECH) were sequentially deparaffinized with xylene and rehydrated through graded alcohol incubations. Sections were then subjected to antigen retrieval using 1 × citrate buffer (Beyotime) in a microwave for 20 min before blocking endogenous peroxidase activity with 3% hydrogen peroxide (Beyotime). Thereafter, the sections were blocked with 10% goat serum at room temperature for 30 min, incubate overnight with primary antibodies OTUD7B (abcam, diluted 1:200), cleaved caspase-3 (CST, diluted 1:400) at 4 °C, and secondary antibodies (Beyotime) at room temperature for 50 min. Immune complexes were revealed using DAB solution (Beyotime), with staining developed in the dark at room temperature before counterstaining with hematoxylin (Beyotime).

### Xenograft models

Six-week-old BALB/c-nude mice weighing between 16-18 g were randomly divided into groups of five animals and allowed to acclimatize for one week before subcutaneously injecting the indicated cells (5 × 10^6^ logarithmic growth phase cells in 200 μL PBS) into the right axilla of each mouse. Once subcutaneous tumors were observed, tumor sizes were estimated every 7 days using caliper measurements, with volumes calculated using the formula V = π/6 × L (length) × W (width) × H (height). After four weeks, mice were sacrificed by cervical dislocation and tumor tissues excised, weighed, and imaged. All animal experiments were conducted with the approval of the Animal Research Ethics Committee of Zhengzhou University (ZZU-LAC20220315[06]).

### Statistical analysis

All experimental data in this study were independently repeated three times and all statistical analyses were performed using GraphPad Prism 8.0 software (GraphPad Software, Inc.). Quantitative data are presented as mean ± standard deviation (Mean ± SD). Comparisons between two groups were made using independent sample t-tests, while multiple group comparisons used one-way ANOVA tests. Pearson correlation coefficient calculations were used to examine associations between variables. P values less than 0.05 were considered to indicate statistically significant findings with p-values less than or equal to 0.05, 0.01, and 0.001 denoted by *, **, and ***, respectively.

## Supplementary Material

Supplementary figures and tables.

## Abbreviations

PTMs: post-translational modifications
DUBs: deubiquitinases
OTUD7B: OTU domain-containing protein 7
HCC: hepatocellular carcinoma
ChIP: chromatin immunoprecipitation
UPS: ubiquitin-proteasome system
OTU: ovarian tumor-related proteases
IP: immunoprecipitation
IHC: immunohistochemistry
KO: knockout
PFTα: pifithrin-α hydrobromide
ALP: autophagy-lysosome pathway
CQ: chloroquine
BafA1: bafilomycin A1
CHX: cycloheximide
CPTAC: Clinical Proteomic Tumor Analysis Consortium
CCK-8: cell counting kit-8
EdU: 5-Ethynyl-2'-deoxyuridine
CDX: cell line*-*derived tumor xenograft
GOF: gain of function
Z-VAD: Z-VAD-FMK
Nec-1: necrostatin-1
Ferr-1: ferrostatin-1
TTM: tetrathiomolybdate
CYCS: cytochrome c
LOF: loss of function
RA: retinoic acid
ATO: arsenic trioxide
STR: short tandem repeat
OD: optical density
