# Supplementary material for: OTUD7B is a new deubiquitinase targeting p53
Source: Theranostics. 2025 Jan 13;15(6):2121–38. doi: 10.7150/thno.103012 (PMC11840744; doi:10.7150/thno.103012)
Supplement: Supplementary file 1 — Supplementary figures and tables. [file thnov15p2121s1.pdf]

Supplementary Materials for

OTUD7B is a new deubiquitinase targeting p53

Caoyuan Ding[1,2#], Leixi Cao[1,#], Ruijie Wang[1], Qichen Wu[1], Mengfan Li[1], Jinjing Zhang[1], Rick F. Thorne[1], Jinming Li[1], Jianli Ma[3,*], Mian Wu[1,*], Shundong Cang[1,*]

[1] Translational Research Institute, People's Hospital of Zhengzhou University, 450003 Zhengzhou, Henan, China.

[2] Department of Pathophysiology, School of Basic Medical Sciences, Zhengzhou University, 450001 Zhengzhou, Henan, China.

[3] Department of Radiation Oncology, Harbin Medical University Cancer Hospital, 150081 Harbin, Heilongjiang, China.

#These authors contributed equally to this work.

*Correspondence: 943328544@qq.com (J.L.M.) or wumian@ustc.edu.cn (M.W.) shundongcang@zzu.edu.cn (S.D.C)

Table S1. Reagents

REAGENT	Catalog #	Company
DMEM	12800082	Gibco
PageRuler Prestained Protein Ladder	26616	Thermo Fisher
opti-MEM	51985091	Thermo Fisher
0.25% Trypsin-EDTA	25200072	Thermo Fisher
SuperSignal™ West Pico PLUS	34580	Thermo Fisher
Embryonic Stem Cell FBS	16141-079	Gibco
Ampicillin	HY-B0522	MCE
Ponceau S	6226-79-5	Sangon Biotech
4% paraformaldehyde	FB002	Invitrogen
Pierce Protein A/G UltraLink Resin	53133	Thermo Fisher
Typtone	A650217-0500	Sangon Biotech
Yeast Extract	A610961-0500	Sangon Biotech
RNase Inhibitor	A005649-0100	Sangon Biotech
Sodium Pyruvate	11360070	Gibco
Penicillin-Streptomycin, liquid	15140148	Gibco
MG132	M8699	Sigma-Aldrich
Cycloheximide (CHX)	239763-M	Sigma-Aldrich
Anti-FLAG® M2 Magnetic Beads	M8823	Sigma-Aldrich
puromycin	HY-B1743A	MCE
Hematoxylin Staining Solution	c0107	Beyotime
Improved Citrate Antigen Retrieval Solution	P0083	Beyotime
DAPI/Hoechst	P0131	Beyotime
ProLong Diamond	P36961	Invitrogen
polybrene	C0351	Beyotime
EDU stainning	CX003	Epizyme
2xTaq PCR mix	P212-01	Vazyme
Z-VAD-FMK	HY-16658B	MCE
Necrostatin-1	HY-15760	MCE
Disulfiram	HY-B0240	MCE
Ferrostatin-1	HY-100579	MCE
Chloroquine (CQ)	HY-17589A	MCE
Bafilomycin A1 (BafA1)	HY-100558	MCE
Tetrathiomolybdate (TTM)	323446-1G	Sigma-Aldrich
Lipofectamine 2000	11668019	Invitrogen
TRIZOL	AM9738	Invitrogen

T4 DNA ligase	2011A	TAKARA
PrimeScript^{TM} RT reagent Kit	RR037A	TAKARA
TB Green® Premix Ex Taq™ II	RR820A	TAKARA
Doxorubicin	HY-15142A	MCE
Cell Counting Kit-8	CK04	Dojindo
Annexin V-FITC/PI staining kit	BD	556547
SimpleChIP® Plus Enzymatic Chromatin IP Kit	9005	Cell Signaling Technology
Nutlin-3	HY-50696	MCE
Pifithrin-α hydrobromide (PFTα)	HY-15484	MCE

Table S2. Antibodies

ANTIBODY	Catalog #	Company
β-actin	66009-1-Ig	Proteintech
GAPDH	60004-1-Ig	Proteintech
OTUD7B	16605-1-AP	Proteintech
OTUD7B	ab238678	abcam
p53	sc-126	Santa Cruz
BAX	50599-2-Ig	Proteintech
Bcl-2	25614-1-AP	Proteintech
BAD	10435-1-AP	Proteintech
Bcl-xL	26967-1-AP	Proteintech
PUMA	55120-1-AP	Proteintech
Mdm2	sc-965	Santa Cruz
PARP1	sc-8007	Santa Cruz
p21	ab109520	abcam
Ubiquitin	3936S	Cell Signaling Technology
Caspase-3	9662S	Cell Signaling Technology
Cleaved caspase-3	9661S	Cell Signaling Technology
Flag-TAG	F3040	Sigma
HA-TAG	H9658	Sigma
GFP-TAG	invitrogen	MA5-15256
Anti-mouse IgG	7076	Cell Signaling Technology
Anti-rabbit IgG	7074	Cell Signaling Technology

Table S3. shRNA primer sequences

shRNA sequences	Primer sequence (5'-3')
sh-OTUD7B-1	ACGTCTTTGTCCTTGCTCA
sh-OTUD7B-2	GTCCGATTGGCCAGTGTAA
sh-p53	CGGGTAGATTACCACTGGAGTCGGATCCGACTCCA GT GGTAATCTACTTTTTG

Table S4. Guide RNA oligonucleotide primers

Guide RNA Primers	Primer sequence (5’-3’)
gRNA-OTUD7B-F1	CACCGGTTACCACCCCCCTACCGAG
gRNA-OTUD7B-R1	AAACCTCGGTAGGGGGGTGGTAACC
gRNA-OTUD7B-F2	CACCGTAGGAGGCCCATAGTCGTCG
gRNA-OTUD7B-R1	AAACCGACGACTATGGGCCTCCTAC
gRNA-p53-F1	CACCGTCGACGCTAGGATCTGACTG
gRNA-p53-R1	AAACCAGTCAGATCCTAGCGTCGAC
gRNA-p53-F2	CACCGCTGTGAGTGGATCCATTGGA
gRNA-p53-R2	AAACTCCAATGGATCCACTCACAGC

Table S5. qRT-PCR oligonucleotide primers

qRT-PCR Primers	Primer sequence (5’-3’)
qRT-β-actin-F	GACCTGACTGACTACCTCATGAAGAT
qRT-β-actin-R	GTCACACTTCATGATGGAGTTGAAGG
qRT-OTUD7B-F	GCTGGAAACCTACCCCCATC
qRT-OTUD7B-R	CATTTCCAGGGGGTGCTCAT
qRT-p53-F	CCCAAGCAATGGATGATTTGA
qRT-p53-R	GGCATTCTGGGAGCTTCATCT
qRT-p21-F	CCTGCCCAAGCTCTACCTT
qRT-p21-R	AAGGCAGGAGATGTAGAGC
qRT-BAX-F	CCCCCGAGAGGTCTTTTTCC
qRT-BAX-R	GAGAGGAGGCCGTCCCAA

Table S6. Recombinant DNA constructs

Recombinant DNA	Source	Identifier
pCMV-HA-OTUD7B	This paper	N/A
pcDNA3.1-OTUD7B-3 × Flag	This paper	N/A
pCMV-HA-OTUD7B-C194S	This paper	N/A
Flag-p53	This paper	N/A
Flag-p53-R175H	This paper	N/A
Flag-p53-G279E	This paper	N/A
GFP-Mdm2	Yide Mei	N/A
His-Ub	This paper	N/A
Flag-p53-△TAD	This paper	N/A
Flag-p53-△PRD	This paper	N/A
Flag-p53-△DBD	This paper	N/A
Flag-p53-△TD_CTD	This paper	N/A
Flag-p53-TAD+TD_CTD	This paper	N/A
Flag-p53-PRD+TD_CTD	This paper	N/A
Flag-p53-DBD	This paper	N/A
Flag-p53-TD_CTD	This paper	N/A

Table S7. CHIP primer sequences

CHIP primers	Primer sequence (5’-3’)
OTUD7B promoter (-959 to -1205)-F	AGTGACAAATGGCCAAAGAACT
OTUD7B promoter (-959 to -1205)-R	GCCCATGTGTATCCAGCCTT
p21 promoter (-43 to +62)-F	GTGGCTCTGATTGGCTTTCTG
p21 promoter (-43 to +62)-R	CTGAAAACAGGCAGCCCAAG

A

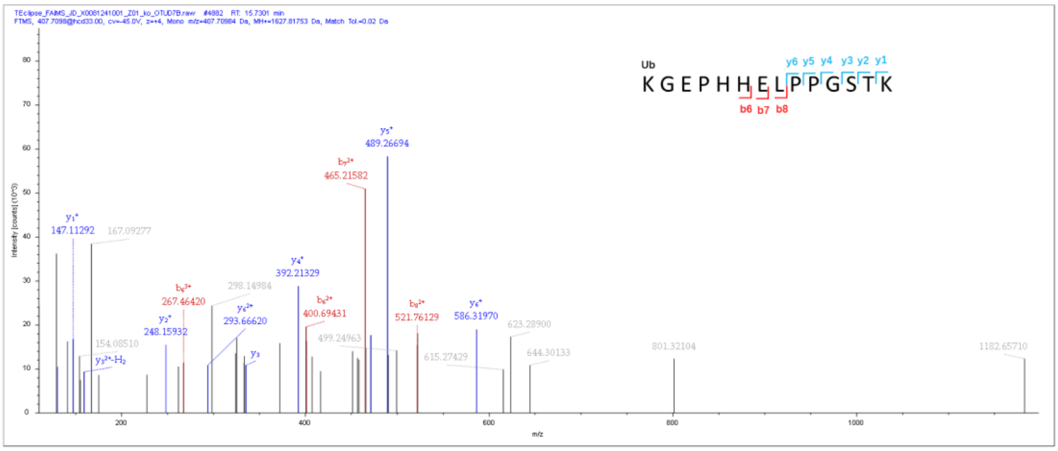

Figure S1. Identification of the major ubiquitination site in p53 targeted by OTUD7B.

A. Cell lysates from WT and OTUD7B-KO HepG2 cells were immunoprecipitated with antibodies against p53 and the samples subjected to mass spectrographic analysis to identify ubiquitination sites affected by OTUD7B expression. The panel shows the MS/MS spectra of one p53 peptide differentially recovered with ubiquitination modifications (sites highlighted in red).

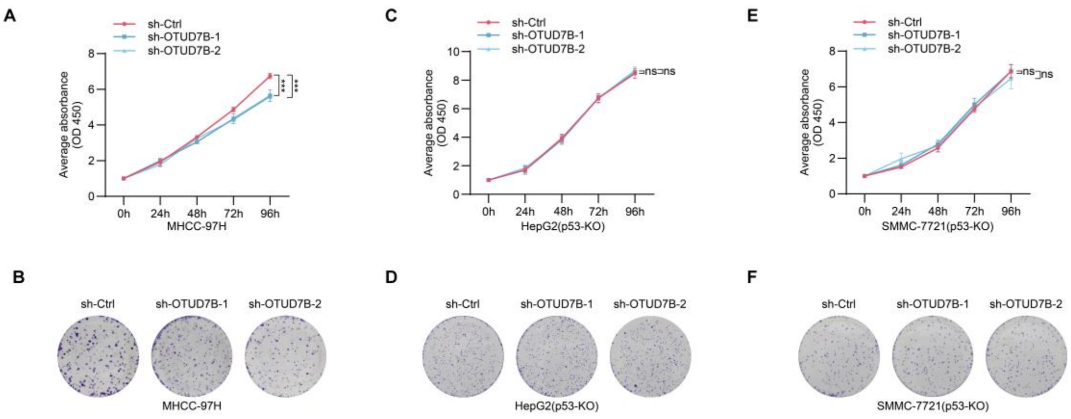

Figure S2. OTUD7B regulates HCC cell proliferation.

A-B. *TP53*-R249S mutant MHCC-97H cells were subject to cell proliferation (D) and clonogenic growth (E) assays as per (Figure 2A-B) after transduction with either control shRNA or two independent OTUD7B-shRNAs.

C-F. HepG2 (p53-KO) and SMMC-7721 (p53-KO) cells were subject to cell proliferation (C-E) and clonogenic growth (D-F) assays as per (Figure 2A-B) after transduction with either control shRNA or two independent OTUD7B-shRNAs.

Data information: The experiments were repeated three times.

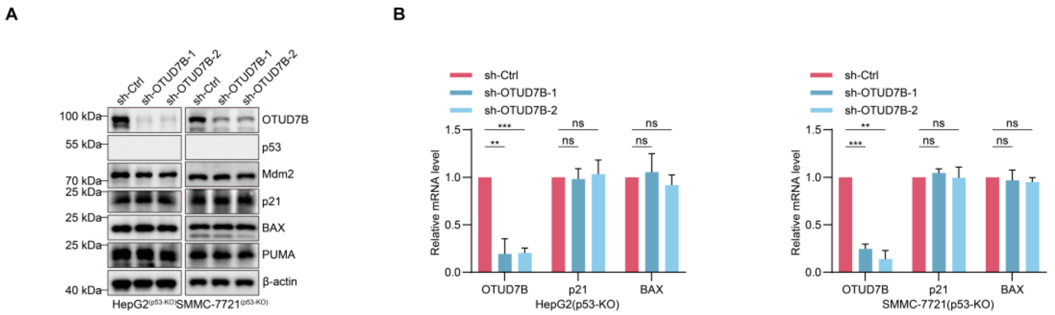

Figure S3. OTUD7B induces apoptosis in HCC cells.

A. Western blot analysis of p53, Mdm2, p21, BAX, PUMA levels in HepG2 (p53-KO) and SMMC-7721 (p53-KO) cells transduced with sh-Ctrl or two independent-shRNAs targeting OTUD7B.

B. QPCR analysis of OTUD7B, p21, BAX levels in HepG2 (p53-KO) and SMMC-7721 (p53-KO) cells transduced with sh-Ctrl or two independent-shRNAs targeting OTUD7B.

Data information: The experiments were repeated three times.
